# Chronic Granulomatous Disease: The European Experience

**DOI:** 10.1371/journal.pone.0005234

**Published:** 2009-04-21

**Authors:** J. Merlijn van den Berg, Elsbeth van Koppen, Anders Åhlin, Bernd H. Belohradsky, Ewa Bernatowska, Lucien Corbeel, Teresa Español, Alain Fischer, Magdalena Kurenko-Deptuch, Richard Mouy, Theoni Petropoulou, Joachim Roesler, Reinhard Seger, Marie-José Stasia, Niels H. Valerius, Ron S. Weening, Baruch Wolach, Dirk Roos, Taco W. Kuijpers

**Affiliations:** 1 Sanquin Research, and Landsteiner Laboratory, Emma Children's Hospital, Academic Medical Centre, University of Amsterdam, Amsterdam, The Netherlands; 2 The Department of Clinical Science and Education, Sodersjukhuset, Sachs' Children's Hospital, Karolinska Institutet, Stockholm, Sweden; 3 Dr v. Haunersches Kinderspital, Ludwig Maximilians University, Munich, Germany; 4 Department of Immunology, The Children's Memorial Health Institute, Warsaw, Poland; 5 Department of Pediatrics, University Hospital, Leuven, Belgium; 6 Immunology Department, Vall d'Hebron Hospital, Barcelona, Spain; 7 Université René Descartes-Paris 5, Hôpital Necker-Enfants Malades, Paris, France; 8 Unité d'Immunologie et d'Hématologie Pédiatrique, Hôpital Necker-Enfants Malades, Paris, France; 9 1st Department of Pediatrics, University of Athens, Aghia Sophia Children's Hospital, Athens, Greece; 10 Department of Pediatrics, University Clinic Carl Gustav Carus, Dresden, Germany; 11 Division of Immunology/Hematology/BMT, University Children's Hospital, Zurich, Switzerland; 12 Centre diagnostic et recherche sur la granulomatose septique, TIMC/Imag, UMR CNRS 5525, Université Joseph Fourier, CHU de Grenoble, Grenoble, France; 13 Department of Pediatrics, Copenhagen University Hospital Hvidovre, Hvidovre, Denmark; 14 Department of Pediatrics and Laboratory for Leukocyte Functions, Meir Medical Center, Kfar Saba, Israel; Duke University, United States of America

## Abstract

CGD is an immunodeficiency caused by deletions or mutations in genes that encode subunits of the leukocyte NADPH oxidase complex. Normally, assembly of the NADPH oxidase complex in phagosomes of certain phagocytic cells leads to a “respiratory burst”, essential for the clearance of phagocytosed micro-organisms. CGD patients lack this mechanism, which leads to life-threatening infections and granuloma formation. However, a clear picture of the clinical course of CGD is hampered by its low prevalence (∼1∶250,000). Therefore, extensive clinical data from 429 European patients were collected and analyzed. Of these patients 351 were males and 78 were females. X-linked (XL) CGD (gp91*^phox^* deficient) accounted for 67% of the cases, autosomal recessive (AR) inheritance for 33%. AR-CGD was diagnosed later in life, and the mean survival time was significantly better in AR patients (49.6 years) than in XL CGD (37.8 years), suggesting a milder disease course in AR patients. The disease manifested itself most frequently in the lungs (66% of patients), skin (53%), lymph nodes (50%), gastrointestinal tract (48%) and liver (32%). The most frequently cultured micro-organisms per episode were *Staphylococcus aureus* (30%), *Aspergillus spp.* (26%), and *Salmonella spp.* (16%). Surprisingly, *Pseudomonas spp.* (2%) and *Burkholderia cepacia* (<1%) were found only sporadically. Lesions induced by inoculation with BCG occurred in 8% of the patients. Only 71% of the patients received antibiotic maintenance therapy, and 53% antifungal prophylaxis. 33% were treated with γ-interferon. 24 patients (6%) had received a stem cell transplantation. The most prominent reason of death was pneumonia and pulmonary abscess (18/84 cases), septicemia (16/84) and brain abscess (4/84). These data provide further insight in the clinical course of CGD in Europe and hopefully can help to increase awareness and optimize the treatment of these patients.

## Introduction

Chronic granulomatous disease (CGD) is an uncommon inherited immunodeficiency, occurring in about one in 250,000 individuals. Although the genetic basis for this disease is well-known, the expected clinical course and outcome have only partially been defined, owing to its sporadic occurrence [1]–[3]. There are extreme differences in presentation between patients, varying form a relatively mild presentation late in life to fatal septicemia in infancy. This renders it difficult for clinicians to inform CGD patients and their parents what to expect, and to find a proper balance between risks, costs and benefits of various treatments, ranging from long-term prophylactic antibiotic medication to stem cell transplantation and gene therapy [4].

CGD is caused by a defect in the burst of oxygen consumption that normally accompanies phagocytosis in myeloid cells (*i.e.* neutrophils, eosinophils, monocytes, and macrophages). The “respiratory burst” involves the catalytic conversion of molecular oxygen to the oxygen free-radical superoxide (O_2_^−^), which in turn gives rise to hydrogen peroxide (H_2_O_2_), hypochlorous acid (HOCl), and hydroxyl radical (^.^OH). These oxygen derivatives play a critical role in the killing of certain pathogenic bacteria and fungi. As a result of the failure to mount a respiratory burst in their phagocytes, the majority of CGD patients suffers from severe recurrent infections and also from dysregulated Th-17-lymphocyte-controled inflammation [5] Therefore, CGD patients can develop diffuse granulomas that can become sufficiently large to cause obstructive or painful symptoms in the esophagus, stomach, ureters, or urinary bladder, or dysfunctional disorders secondary to extensive fibrosis of the different systems (pulmonary, gastrointestinal, genitourinary, central nervous system) [6]–[9]. Also, autoimmune phenomena have been reported to occur at an increased incidence rate in CGD patients and in female X-linked carriers [10]–[12].

The enzyme that catalyzes the respiratory burst, the leukocyte NADPH oxidase, consists of subunits, four of which are important for CGD (designated *phox* for *ph*agocyte *ox*idase): gp91^phox^ (or Nox2) and p22^phox^, located in membranes, as well as two cytosolic oxidase components, p47^phox^ and p67^phox^. CGD is caused by a defect in any of these four components. Mutations in the gp91^phox^ gene (*CYBB* on chromosome Xp21.1) cause the X-linked recessive form of the disease that affects the majority of CGD patients (∼70%). As expected from the genetics, the overwhelming majority of X-linked patients are males. The remaining 30% of cases has inherited the disease in an autosomal recessive manner, in which males and females are equally affected. These patients have mutations in the genes encoding p47^phox^ (*NCF1* on chromosome 7q11.23), p67^phox^ (*NCF2* on chromosome 1q25), or p22^phox^ (*CYBA* on chromosome 16q24). In contrast to p47^phox^ deficiency [13], most CGD patients with any of the other three forms have mutations unique to their families [14].

Apart from the description of several smaller cohorts, the first characterization of a large group of CGD patients was published by Winkelstein et al., who reported on a national American registry of 368 patients [1]. To further improve knowledge of the course of CGD, clinical data from European patients were collected. We now report our findings in 429 European patients, the largest cohort of CGD patients to date.

## Methods

In most European countries, diagnostic tests for CGD are traditionally performed by a small number of specialized institutes. The clinical heads of these institutes were asked to collect data from the medical charts of their CGD patients, by means of an extensive questionnaire. (Participating institutes: Emma Children's Hospital, Academic Medical Centre, Amsterdam, The Netherlands; Sachs' Children's Hospital, Karolinska Institutet, Stockholm, Sweden; Dr v. Haunersches Kinderspital, Munich, Germany; The Children's Memorial Health Institute, Warsaw, Poland; Department of Pediatrics, University Hospital, Leuven, Belgium; Vall d'Hebron Hospital, Barcelona, Spain; Université René Descartes-Paris 5, Hôpital Necker-Enfants Malades, Paris, France; Unité d'Immunologie et d'Hématologie Pédiatrique, Hôpital Necker-Enfants Malades, Paris, France; University Clinic Carl Gustav Carus, Dresden, Germany; University Children's Hospital, Zurich, Switzerland; Centre diagnostic et recherche sur la granulomatose septique, Université Joseph Fourier, Grenoble, France; Dept of Pediatrics, Copenhagen University Hospital Hvidovre, Denmark; Department of Pediatrics and Laboratory for Leukocyte Functions, Meir Medical Center, Kfar Saba, Israel). Approval was obtained from the medical ethical committee of the Academical Medical Center in Amsterdam, The Netherlands. Informed consent forms were signed by those patients who were alive and not lost to follow up. The answers on the completed questionnaire of each individual patient were subsequently translated to a database and analyzed. Prior to analysis, the patients' information was completely anonymized. The data were collected between the years 2000–2003.

In total, questionnaires of 446 patients were sent in, of which 17 were eventually omitted because of incomplete data, resulting in 429 individuals analyzed. The oldest clinical information dated from 1954, the most recent from 2002.

The diagnosis of CGD was established in all 429 patients by means of several well-described diagnostic tests, such as the nitroblue tetrazolium assay (NBT) or the dihydrorhodamine-1,2,3 (DHR) test [15]–[16]. In the majority of patients, the diagnosis was confirmed by Western blot, showing the absence of one of the NADPH oxidase components, and DNA analysis on blood samples of the patient or his relatives [17]. Determination of the CGD subtype was also performed by means of Western blot and/or DNA analysis. However, in some cases, deceased relatives of proven CGD patients, who had previously died from unexplained overwhelming infection or granulomatous disease, were included (i.e., maternal uncles or siblings from affected “established” patients).

Of female patients in which the diagnosis CGD had been established by adequate laboratory analysis, but the subtype had not been established by Western blot or DNA sequencing, it was assumed they were suffering from an autosomal recessive form of the disease (20 cases). Deficiency of gp91^phox^ in females, leading to clinical CGD, can only be caused by extremely unfavorable lionization [18], which is very rare. In 17 male patients, the exact subtype of CGD was not established, but either the presence of gp91^phox^ was shown by Western blot, or the family history strongly suggested an autosomal recessive mode of inheritance. This led to the conclusion that these patients also suffer from AR-CGD.

## Results

### 1. Population

The origin of the patients was categorized according to the country of residence or alternatively to the country of origin of the parents when this information was provided. Of 438 patients, the largest group were French (84), followed by Germans (69), Dutch (62), immigrants from Arab/ North African descent (38), Poles (30), Spaniards (25), Swedes (24), Swiss (24), Danes (22), Israeli/Jewish (16), immigrants from Turkey (12), (former) Yugoslavs (8), Belgians (6), immigrants from miscellaneous Asian countries (6), Italians (2) and Austrians (1) (Table 1). Patients in the Arab/North African group were living in several European countries, but also included 6 individuals form Arab descent living in Israel, the remainder of the Israeli group being of Jewish origin.

**Table 1:** The cohort of CGD patients divided according to nationality/ ethnic background and type of CGD.

Country/ Region of Origin	Total	CGD type	
		*X-linked*	*AR* *
France	**84**	75 *(89%)*	9 *(11%)*
Germany	**69**	54 *(78%)*	15 *(22%)*
The Netherlands	**62**	43 *(69%)*	19 *(31%)*
Arab/North African	**38**	11 *(29%)*	27 *(71%)*
Poland	**30**	21 *(70%)*	9 *(30%)*
Spain	**25**	16 *(64%)*	9 *(36%)*
Sweden	**24**	12 *(50%)*	12 *(50%)*
Switzerland	**24**	19 *(79%)*	5 *(21%)*
Denmark	**22**	12 *(55%)*	10 *(45%)*
Israeli/Jewish	**16**	9 *(56%)*	7 *(44%)*
Turkey	**12**	4 (*33%)*	8 *(67%)*
Former Yugoslavia	**8**	7 *(88%)*	1 *(12%)*
Belgium	**6**	5 *(83%)*	1 *(17%)*
East and South Asia	**6**	1 *(17%)*	5 *(83%)*
Italy	**2**	1	1
Austria	**1**	0	1
**Total**	**429**	**290 ** ***(67%)***	**139 ** ***(33%)***

* AR = autosomal recessive.

Conspicuously absent in this database were patients from the United Kingdom, Ireland and Italy. In these countries national registries of the clinical data from CGD patients were being constructed independently, the data of which have recently been published separately [2]–[3]. One remarkable observation is the high prevalence of the autosomal recessive forms of CGD in immigrant groups from Arab/ North African countries (71%) and Turkey (67%), reflecting consanguineous marriage practices common within these communities.

Of the 429 patients, 351 were male (82%) and 78 female (18%), including 6 heterozygous female carriers with an extremely unfavorable lyonization (Table 2) [19]. This corresponds with the data known from earlier publications [1].

**Table 2:** Distribution of different subtypes of CGD according to sex.

	Male	Female	Total
*X-linked*	284 *(81%)*	6 *(8%)* *	290 *(67%)*
*AR*	67 *(19%)*	72 *(92%)*	139 *(33%)*
***Total***	**351 ** ***(82%)***	**78 ** ***(18%)***	**429**

* Female X-linked patients are extremely lyonized heterozygous carrriers.

Within the AR-CGD subgroup, deficiency of p47*^phox^* was most prevalent (49%), as was the case in the American CGD cohort [1], followed by p22*^phox^* (16%) and p67*^phox^* (8%) (Table 3).

**Table 3:** Different subtypes of autosomal recessive CGD.

AR type	Male	Female	Total
*p47*	38 *(57%)*	31 *(42%)*	69 *(49%)*
*p22*	10 *(15%)*	12 *(16%)*	22 *(16%)*
*P67*	2 *(3%)*	9 *(13%)*	11 *(8%)*
*Unknown*	17 *(25%)*	20 *(28%)*	37 *(27%)*
***Total***	**67**	**72**	**139**

### 2. Clinical course

**Figure 1:**
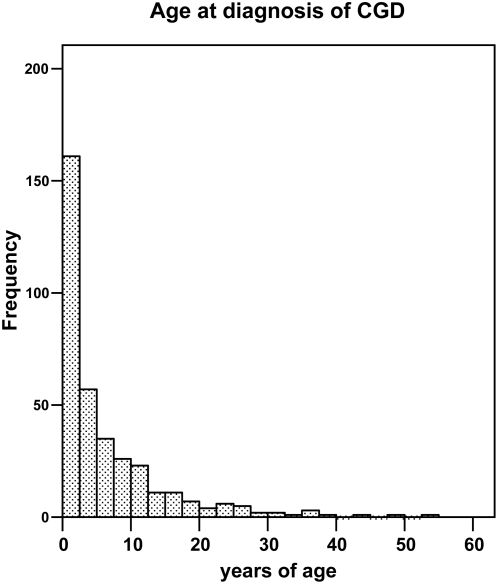
The age at diagnosis of CGD, irrespective of the subtype. Each column represents 2.5 years.

**Figure 2:**
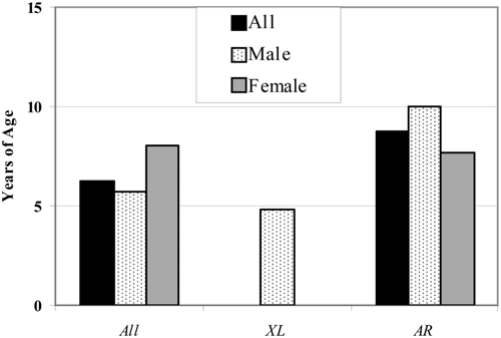
Average Age at Establishment of Diagnosis “CGD”.

The diagnosis CGD was established usually early in life (Figure 1). However, the mean age at diagnosis (Figure 2; Table 4) was higher in the AR CGD group (8.8 years) than in X-linked CGD (4.9 years) (p<0.001, as assessed by Mann-Whitney U analysis). This probably reflects a milder phenotype of AR-CGD, and hence an extensive immunological work-up at a later age, and not a bias towards earlier screening of boys for CGD. The mean age at diagnosis of AR-CGD in boys was even higher than in girls (10.0 vs 7.7 years). Another possible explanation could be more adequate screening of possibly affected boys in extended families with X-linked CGD, leading to very early diagnosis (*e.g.* at birth) before any signs of the disease have become manifest.

**Table 4:** Clinical course in CGD.

	XL	AR
*Avg. age at diagnosis (years)*	4.9 (range: a.n+.- 43 yrs)	8.8 (range: a.n.- 54 yrs)
*Deceased* ¶	64/279 (23%)	20/136 (15%)
*Median age at time of death (years)*	8.8 (range: 0.16–34.5)	10.4 (range: 1.1–54.8)
*Mean survival time (years)*	37.8	49.6

Clinical course in CGD patients is milder in AR patients, as reflected by a later age at diagnosis and longer survival.

+ a.n. = antenatal.

¶ 14 out of 429 patients were excluded because reliable data on survival were lacking.

At the time of data collection 84 patients had died (20%). Of 331 individuals, the last known date when the patient was reported to be alive was used for statistical analysis. 14 patients were excluded because of the lack of reliable data.

**Figure 3:**
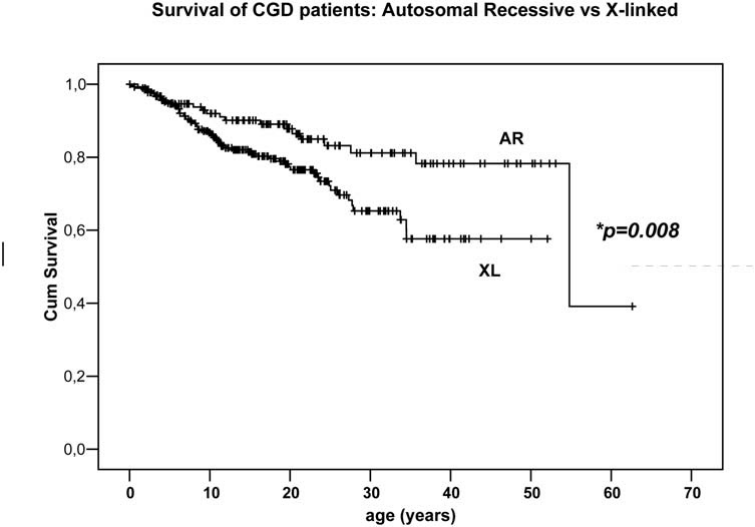
Cumulative survival of AR patients compared to XL patients. *Log rank statistics for difference in survival between X-linked and AR: 7.01 (p = 0.0081).

Clearly, AR patients were not only diagnosed later, but a higher percentage had survived up to the time of inclusion in this cohort. Also, the age at death and the mean survival time were much higher in the AR group (Table 4). Statistical analysis, as reflected in Figure 3, showed that this difference was highly significant (p = 0.0081). These data confirm the widely held conviction that AR-CGD follows a milder course than XL-CGD.

### 3. Clinical symptoms

The most prevalent site of disease, be it infectious or granulomatous, were the lungs, followed by skin/subcutis, lymph nodes, gastro-intestinal tract and liver (Table 5). These findings confirm earlier reports and general assumptions on the clinical presentation of CGD [1]–[3]. From the data collected, it was not possible to adequately determine the first symptom or site of disease at presentation.

**Table 5:** Site of disease.

Site of Disease	Number of episodes	Number of patients with ≥1 episode	% of patients with ≥1 episode
Lung	634	284	**66%**
Skin/ Subcutis	341	229	**53%**
Lymph node	622	213	**50%**
Gastro-intestinal	643	208	**48%**
Liver	240	138	**32%**
Kidney/ Urinary tract	139	95	**22%**
Septicaemia	111	85	**20%**
Ear	84	62	**14%**
Bone	84	56	**13%**
Eye	68	46	**11%**
Joint	35	31	**7%**
Brain	34	31	**7%**
Autoimmunity- Rheumatology	26	26	**6%**

The total number of reported episodes in all patients is compared to the number of patients who has suffered from ≥1 episode in absolute numbers and percentage of the total group (n = 429).

Pulmonary involvement (634 episodes in 284 patients, 66% of the total population) most frequently consisted of pneumonia (597 episodes) and rarely of lung abscess (26 episodes in 24 patients) (Table 6). Pulmonary granulomatous disease, as defined by a histological diagnosis and negative culture, was only proven in 11 episodes in 10 patients. This presentation of CGD is sometimes initially confused with mycobacterial infection or sarcoidosis [9]. The most frequently isolated microorganism was *Aspergillus spp.* (111 cases), other pathogens were rarely seen. Sporadic cases included *Cephalosporium* (2×), *Streptococcus pneumoniae* (2×), *Scedosporium* [20] (1×), *Paecilomyces* [21] (1×), *Phialophora richardsiae* (1×), *Rhodococcus equi* [22] (1×) and *Staphylococcus epidermidis* (1×). Some of these rare pathogens have been described in earlier reports to cause infection in CGD.

**Table 6:** Pulmonary involvement and cultured microorganisms.

	Pneumonia	Lung Abscess	Granulomatous	Total
***Number of episodes***	597	26	11	634
	***Cultured micro-organism:***			
*Aspergillus spp.*	111 (18%)		*Klebsiella spp.*	4
*Candida spp.*	10 (2%)		*Pseudomonas spp.*	3
*S. aureus*	10 (2%)		*Streptoccus viridans*	3
*H. influenzae*	6 (<1%)		*Actinomyces*	2
*M. tuberculosis*	5		*Acremonium*	2
*Atypical Mycobacteria*	3		*Misc.*	9
*Nocardia spp.*	4			
*Negative culture/ not done*	454 (72%)			

Pulmonary involvement and cultured microorganisms (in total 634 episodes in 284 patients). Percentages are the number of episodes with a positive culture of a certain pathogen divided by the total number of reported episodes.

Remarkably rare in this cohort was *Burkholderia cepacia*, a pathogen frequently described to have caused fulminant pneumonia in CGD [1], [23]. The low number of positive cultures for *Pseudomonas spp*. (3) excludes the possibility that a significant number of *B. cepacia* isolates could have been described as *Pseudomonas* (*B. cepacia* was previously known as *Pseudomonas cepacia*). However, in the majority of episodes the causative pathogen could not be isolated.

After the lungs, the skin is the most frequently affected site of disease (Table 7). The etiology is diverse, ranging from abscesses in the skin to aseptic granulomas. Discoid lupus erythematodes (DLE) is not included in this section (*vide infra*). When cultures were performed, *S. aureus* was most prominent. Occasional pathogens found in skin abscesses (131 cases) included *S. epidermidis* (3×), *Serratia marcescens* (2×), *Proteus mirabilis* (2×), *Escherichia coli* (2×) and *Klebsiella pneumoniae* (1×).

**Table 7:** Diseases of the skin in CGD and causative pathogens.

	Abscess	Dermatitis	Acne	Furunculosis	BCGitis	Granuloma	Total
***Number of episodes***	131	101	29	39	24	11	341
***Number of patients with ≥1 episode***	74	71	25	21	24	10	229
***% of patients with ≥1 episode***	17%	17%	7%	5%	6%	2%	**53%**
	***Most frequently cultured microorganisms***						
	***Abscess***			***Dermatitis***		***Furunculosis***	
	*S. aureus (12%)*			*S. aureus (17%)*		*S.aureus (14%)*	
	*Aspergillus spp. (5%)*			*Candida spp. (5%)*			
				*S. epidermidis (5%)*			
				*Serratia marcescens (3%)*			

Vaccination with Bacille Calmette-Guérin (BCG) resulted in subsequent localized skin infection in 24 patients (BCG-itis). In addition, BCG was isolated from lymph nodes of 10 patients suffering from lymphadenitis (Table 8). Thus, BCGitis occurred in 34/429 patients (8%). There was no association between the occurrence of BCGitis with CGD type (AR or XL), nor with sex. A remarkably high percentage of this group was living in France (22 = 65%). As a whole, the French cohort in this database consisted of 100 individuals, 84 of which were of French ancestry and 16 originated from other countries, mainly from North Africa. Thus, 22% of the French patients developed BCGitis at one point in time during their lives. This group included 18 ethnic French, two Arabs and two individuals of Asian descent, suggesting that not ethnic differences, but rather exposure was the cause of this high incidence. BCG vaccination was given routinely to all French children until recently, whereas other European countries only vaccinate children with an increased risk of being infected with tuberculosis (*e.g.* children of immigrants traveling to their home countries). Susceptibility to *Mycobacterium tuberculosis* or atypical mycobacteria was not clearly increased. Recently, a number of reports, some of which have so far not been published, have claimed there is indeed an increased susceptibility for mycobacterial infection in CGD [24]–[25].

**Table 8:** Lymphadenitis.

Number of episodes	622 in 213 patients		
	***Cultured micro-organism:***		
*S. aureus*	76 (12%)	*B. cepacia*	4
*S. marcescens*	11 (2%)	*Aspergillus spp.*	3
*Post-BCG*	10 (2%)	*Candida spp.*	3
*S. epidermidis*	8 (1%)	*M. tuberculosis*	2
*Klebsiella spp.*	6 (<1%)	*Salmonella spp.*	2
*Atypical Mycobacteria*	5	*Misc.*	4
*Negative culture/ not done*	495 (80%)		

Lymphadenitis and cultured micro-organisms (in total 622 episodes in 213 patients). Percentages are the number of episodes with a positive culture of a certain pathogen divided by the total number of reported episodes.

Again, in lymphadenitis, a frequent manifestation of CGD, in most episodes no causative pathogen was isolated, either because cultures were negative or because a fine needle aspirate or biopsy had not been performed (80%, Table 8). *S. aureus* was cultured most frequently, as was expected. Sporadically, lymphadenitis was caused by *Actinomyces* (1×), *Haemophilus influenzae* (1×), *Philalophora richardsiae* (1×) and *P. mirabilis* (1×). Vaccination with BCG resulted in regional lymphadenitis in 7 individuals (see above).

CGD can manifest itself in the gastrointestinal tract from the oral cavity to the anus (Table 9) [8]. Although gastrointestinal symptoms occurred even more often than pulmonary symptoms (643 *vs* 634 episodes), the number of patients who had suffered from one or more gastrointestinal symptoms was lower (48% *vs* 66%). Apart from non-typhoid *Salmonella spp.* gastroenteritis, no relevant other microorganisms were cultured from stool samples or biopsies. Granulomatous disease was frequently found in the gastrointestinal tract, ranging from esophageal and pyloric obstruction caused by granulomas to granulomatous disease of the colon mimicking the clinical presentation of Crohńs colitis. In 26 out of 66 cases of suspected colitis, the diagnosis was reported to have been confirmed by colonoscopy and histology of biopsies. Appendicitis, although often cited as one of the presenting signs of intestinal granuloma formation in young CGD patients, is rare in this cohort, not exceeding the prevalence in the healthy population.

**Table 9:** Gastrointestinal manifestations of CGD.

	Number of episodes	Number of patients with ≥1 episode	% of patients with ≥1 episode
*Gastroenteritis (50% Salmonellosis)*	140	112	26%
*Peri-anal abscess/ fissure*	145	88	21%
*Diarrhea*	71	55	13%
*Stomatitis*	66	48	11%
*Aphthae*	58	48	11%
*Gingivitis/ Caries*	51	47	11%
*Colitis (38% granulomatous)*	66	40	9%
*Pyloric outlet obstruction*	16	9	2%
*Esophageal granuloma/obstruction*	11	9	2%
*Appendicitis*	7	7	2%
*Pancreatitis*	8	6	1%

240 episodes of liver abscess were seen in 138 patients (32% of total), most frequently caused by *S. aureus* (25%), as expected (Table 10).

**Table 10:** Prevalence of liver abscess and causative micro-organisms.

Number of episodes	240 in 138 patients (32%)		
	***Cultured micro-organism:***		
*S. aureus*	59 (25%)	*Candida spp.*	1 (<1%)
*Aspergillus spp.*	6 (3%)	*Streptococcus milleri*	1
*S. marcescens*	5 (2%)	*Enterobacter cloacae*	1
*Negative culture;*	167 (70%)		

In 23% of the patients involvement of the kidneys or the urinary tract was seen, sometimes leading to renal insufficiency (Table 11). Although infection is the most common manifestation of urinary tract involvement, more serious complications are found when obstruction by granulomata causes hydronephrosis or obstruction of the ureter, bladder or urethra [7], which was found in 6% of the patients.

**Table 11:** Involvement of kidneys and urinary tract (UT).

	Urinary tract infection*	Granulomata (causing UT obstruction)	Renal insufficiency	Total
***Number of episodes***	77	44	18	139
***Number of patients with ≥1 episode***	52	25	18	98
***% of patients with ≥1 episode***	**12%**	**6%**	**4%**	**23%**
	***Most frequently cultured micro-organisms in UTI***			
	*E. coli(13%)*		*Salmonella spp. (4%)*	
	*P. mirabilis (5%)*		*K. pneumoniae (4%)*	

* including three episodes of renal abscess.

Septicemia was seen in 20% of the patients (111 episodes in 85 patients), most frequently caused by *Salmonella spp.*, followed by *S. aureus* (Table 12). Miscellaneous causative bacteria included *Listeria monocytogenes*, *Propionibacterium acnes*, *P. mirabilis* and *Yersinia enterocolitica* (all 1 case).

**Table 12:** Prevalence and causes of septicemia.

Number of episodes	111 episodes in 85 patients		
	***Cultured micro-organism:***		
*Salmonella spp.*	36 (32%)	*B. cepacia*	3 (3%)
*S. aureus*	11 (10%)	*Pseudomonas spp.*	2 (2%)
*S.epidermidis*	6 (5%)	*K. pneumoniae*	2 (2%)
*Aspergillus spp.*	4 (4%)	*E. coli*	2 (2%)
*Candida spp.*	3 (3%)	*S. marcescens*	1 (1%)
*Streptococcus spp.*	3 (3%)	*Misc.*	5 (5%)
*Negative culture*	23 (21%)		

Otitis media was found in 14% of the patients (84 episodes in 62 patients, not shown). In 89% of the episodes, no cause of the otitis was identified, the remaining episodes being caused by *P. aeruginosa* (5×), *S. aureus* (1×), *Streptococcus viridans* (1×) and *K. pneumoniae* (1×).

Osteomyelitis, seen in 84 episodes in 56 patients (13%), was caused mostly by *Aspergillus spp.*, followed by *Serratia marcescens*, confirming earlier data [1]. Sporadic episodes included *S. epidermidis* (2 episodes), *Providencia* and *Nocardia* (all 1 case) (Table 13). The most frequently affected bones were the ribs (15 episodes), vertebrae (8 episodes), femur (7 episodes), talus (6 episodes) and tibia (5 episodes). The bones of the hand, thought to be a predilection location for osteomylitis in CGD, were only affected in 4 cases in this cohort.

**Table 13:** Prevalence and causes of osteomyelitis.

Number of episodes	84 cases in 56 patients		
	***Cultured micro-organism:***		
*Aspergillus spp.*	*29 (35%)*	*S. epidermidis*	*2 (2%)*
*Serratia spp.*	*7 (8%)*	*Salmonella spp.*	*2 (2%)*
*S. aureus*	*3 (4%)*	*Enterobacter spp.*	*2 (2%)*
*Negative culture; unknown*	*37 (44%)*	*Misc.*	*2 (2%)*

The eyes were involved in 11% of the patients (68 episodes in 46 patients, not shown). Conjunctivitis was the most prevalent (21 cases), without identified organisms, as was the case with blepharitis (5 cases). Chorioretinitis, a well known but not well understood feature also found in other immunodeficiencies, was seen in 8 patients [26].

Septic arthritis accounted for 36 episodes in 32 patients (8%) (table not shown). However, only in the minority of episodes a pathogen was cultured, mostly *S. aureus* (4), followed by *Serratia spp.* (3), *Aspergillus spp.* and *Nocardia* (both 1 episode).

Table 14 shows the prevalence of brain abscesses, a most feared complication of CGD (see below). Thirty-one patients (7%; 34 episodes) developed a brain abscess, mostly caused by *Aspergillus spp.*

**Table 14:** Prevalence and causes of brain abscess.

Number of episodes	34 episodes in 31 patients		
	***Cultured micro-organism:***		
*Aspergillus spp.*	*13 (38%)*	*Klebsiella spp.*	*1 (3%)*
*Salmonella spp.*	*2 (6%)*	*S. aureus*	*1 (3%)*
*Negative culture; unknown*	*17 (50%)*		

With all infectious episodes taken together, the most frequently cultured microorganism was *S. aureus* (30%), followed by *Aspergillus spp.* (26%) and *Salmonella spp* (16%), with all other micro-organisms occurring rather seldom or even sporadically (Table 15). These findings roughly reflect previous data. However, the relatively low number of isolates of *Burkholderia cepacia* is remarkable, seen in the light of earlier reports.

**Table 15:** Most frequently isolated micro-organisms.

Cultured micro-organism	No	% of total
*S. aureus*	210	30%
*Aspergillus spp.*	181	26%
*Salmonella spp.*	110	16%
*Candida spp.*	45	6%
*Serratia spp.*	31	4%
*S. epidermidis*	22	3%
*Klebsiella spp.*	14	2%
*Pseudomonas spp.*	11	2%
*M. tuberculosis*	8	1%
*Atypical Mycobacteria*	8	1%
*H. influenzae*	6	<1%
*B. cepacia*	7	<1%
*Streptoccus spp.*	7	<1%
*Nocardia*	4	<1%
*Misc.*	37	5%
***Total***	**701**	

Manifestations of autoimmunity or autoinflammation have been claimed to be more prevalent in CGD patients than in the normal population. Mothers of boys with X-linked CGD, themselves carriers, are known to be prone to lupus or lupus-like skin lesions, such as discoid lupus erythematodes [12]. In this cohort, signs of autoimmune disease were seen in 26 patients (6%), indeed mostly discoid lupus (18 cases). Other manifestations were sporadic, including rheumatoid arthritis (2 cases), systemic lupus erythematodes, dermatomyositis, sacroiliitis, idiopathic thrombocytopenia and autoimmune hepatitis (all 1 case). Thus, apart from discoid lupus, of which the estimated prevalence in the general population is much lower than in this cohort, other signs of autoimmunity or rheumatic disorders seem to be no more prevalent than in healthy individuals (not shown).

### 4. Prophylactic and curative therapies

Most CGD patients are treated with long-term, low-dose antibiotics and antifungals [27]. In this cohort, 71% of the patients were reported to use antibiotic prophylaxis at at least one point of their clinical history, mostly co-trimoxazole (Table 16). Antifungals had been used by 53%, mostly itraconazole. Interferon-γ by subcutaneous injection up to 3 times a week has been given to many patients after a trial had shown a protective result against infection [28]. A true understanding of the positive effect of IFN-γ on neutrophil biology or innate immunity in general is still lacking. Combined with side effects in some patients, this has prompted many clinicians to administer IFN-γ to some, but not all patients. In this cohort, 33% of all patients had at some point received IFN-γ. Unfortunately, it was not possible from the collected data to link long-term maintenance therapies, be it antibiotics, antifungals or IFN-γ, with the occurrence of infection, complications, or death.

**Table 16:** Prophylactic and curative treatment.

Type of treatment	% Patients who received treatment		
***Antibiotic prophylaxis***	**71%**		
	Of which:	Co-trimoxazole	42%
		Ciprofloxacin	4%
		Clindamycin	3%
		Rifampicin	3%
***Antifungal prophylaxis***	**53%**		
	Of which:	Itraconazole	47%
		Amphotericin B	15%
		Ketoconazole	7%
***Interferon-γ***	**33%**		
***Granulocyte Transfusion***	**7%** (32×)		
***Stem cell transplantation***	**6%** (24×)		

Percentages of patients who received above-mentioned therapies at one point during their lives.

Granulocyte transfusions are increasingly given to CGD patients when traditional therapies fail to resolve life-threatening infections, especially with *Aspergillus spp.* [29]. Seven percent of the patients received a granulocyte transfusion at one point.

A curative therapy for CGD is stem-cell transplantation. However, this treatment has a considerable risk of transplantation-related morbidity and mortality, especially in patients with severely compromised pulmonary function or other debilitating illnesses [30]. In this cohort, 24 patients (6%) had been treated with stem-cell transplantation, of whom 5 had died at the time of inclusion. No patients in this cohort had at the time of inclusion been treated with gene therapy. This treatment had a promising, though transient, clinical effect in some selected patients [31], but insertional mutagenesis also bears new risks.

### 5. Cause of death

As mentioned above, 84 patients had died before the time of inclusion (20%) (Table 4, Table 17). The largest number of patients had died from pneumonia, with a particular increased risk from aspergillosis. Septicemia was the cause of death in another 16 patients. Although *Burkholderia cepacia* did not account for a high number of infectious episodes in this cohort, a relatively high number of patients died from septicemia with this pathogen (3), illustrative of the possible fulminant nature of *Burkholderia cepacia* infection. *Aspergillus spp.* was again the most prominent pathogen to cause death in patients with a brain abscess. Only one patient died of malignancy (malignant melanoma). In this cohort, three patients suffered from a malignant disease, the two others from leukemia/lymphoma. This does not point to a clear, significantly increased risk for malignant disease in CGD patients.

**Table 17:** Cause of death.

Number of deceased patients: 84 (20%)		
***Pneumonia: 18 (21%)***	Of which:	*Aspergillus spp.*: 12
		Bacterial n.o.s*.: 6
***Septicemia: 16 (19%)***	Of which:	n.o.s. :10
		*Burkholderia cepacia*: 3
		*Salmonella spp.*: 2
		*Candida spp.*: 1
***Brain abscess: 4 (5%)***	Of which:	*Aspergillus spp.*: 3
		Bacterial n.o.s.: 1
***Cardiac Failure***	3 (4%)	
***Chronic Lung Disease***	2 (2%)	
***Liver Abscess***	2 (2%)	
***Post BMT***	2 (2%)	
***Malignancy***	1 (1%)	
***Miscelaneous***	5 (6%)	
***Unknown***	31 (37%)	

* n.o.s. = not otherwise specified.

## Discussion

This is the largest cohort of CGD patients described so far. It combines clinical data collected from most of the prominent centers on the European continent that have dealt with the diagnosis and treatment of CGD for several decades. From the huge amount of clinical information collected, the most relevant data are presented here.

It is still not possible to determine the true incidence and prevalence of CGD. From this cohort one would discern that in the Netherlands the prevalence of CGD is high (62 cases on a population of 16 million), and even higher in Denmark (22 cases on a population of 5.5 million). Most likely these numbers are due to a high awareness of the disease. This view is supported by the observation that more cases of CGD are found when laboratory diagnostic tools are improving [3].

A weakness of this cohort is the absence of patients from two large European countries, the UK and Italy [2]–[3]. Also, clinical data from many Central and Eastern European countries have not been added to the present database. Another problem is the quality of older data, a common challenge in the analysis of retrospective databases. In order to improve the clinical data analysis of CGD patients, many data from other countries will have to be added, while the quality of newly added information has to be warranted. These issues will be addressed by a new on-line, prospective, clinical registry for European patients that is currently under construction by the European Society for Immune Deficiencies (ESID).

Because of the retrospective nature of the current database, it was not possible to clearly define at what time point exactly the patients suffered from the different afflictions characteristic of CGD. Thus, the effect of prophylactic treatment could not be analyzed. Although the use of antibiotic prophylaxis in CGD patients is undisputed, with antifungal prophylaxis, side-effects of itraconazole (which is routinely used) and the burden of taking life-long daily drugs will have to be compared to the effect of prompt and effective therapeutic treatment when fungal infection is suspected in patients who do not take antifungal prophylaxis. Newer antifungal drugs such as posaconazole might be more beneficial, as they are believed to cause less side effects. Also, the evidence that interferon-gamma helps prevent serious infection in CGD does not warrant its use in all patients. Again, these are issues that could be addressed better by collecting data in a prospective fashion.

The lack of an adequate time scale also complicates a proper description of the first presenting signs, which would be of great value for the training of physicians in order to recognize CGD and to initiate proper diagnostic testing.

Clinicians have noted, and reported, a milder clinical course for AR CGD patients leading to longer survival as compared to XL CGD patients for many years [32]. Owing to the small number of CGD patients, it has been difficult in the past to substantiate this claim. The data presented in this paper have confirmed earlier reports and show a clear statistically significant difference in life span between the two main types of CGD, although it is important to keep in mind that there have been individual AR-CGD patients with early and extremely severe expression of the disease. It is not clear why AR CGD in general follows a milder course, although residual NADPH-oxidase activity has been shown in p47^phox^ deficiency [33]–[34]. A difference in bactericidal activity of neutrophils from the two groups has so far not been shown. From this cohort, an increased susceptibility to one particular pathogen in XL CGD patients was not apparent.

The isolated micro-organisms roughly correspond to the pathogens known to be important in CGD [1]–[3]. However, many cultures remained negative, perhaps owing to previous long-term antibiotic/antifungal prophylactic therapy. A remarkable finding was the relative paucity of *Burkholderia cepacia* as an important pathogen in CGD. Further analysis of prospectively collected data may clarify whether *Burkholderia cepacia* is indeed less frequently found in CGD in Europe as compared to the United States and the UK [1], [3]. The severity of infection with *Burkholderia cepacia* was examplified by the high percentage of lethal cases. Three patients out of seven established infections with *B. cepacia* had died (Tables 15 and 17). However, as a cause of death, *Aspergillus spp.* stand at the top, warranting an even more aggressive approach to this infection in CGD patients. Most clinical data are from a time when dagnostic tools such as high resolution CT-scans and MRI were not available. Also, many succesful anti-fungals currently in use, such as voriconazole [27], [35] or posaconazole [36], had at the time not yet been developed. Better diagnosis and treatment should lead to a reduction in *Aspergillus*-related mortality in the future.

Our data also confirm an increased susceptibility to BCG-itis in CGD patients, as has been recently described [24], [25]. This newly described feature warrants further investigation. It could be that a hampered oxidative burst in macrophages complicates an adequate clearance of the weak BCG pathogen, thus leading to prolonged inflammatory activity.

Although the median life expectancy in this study was relatively high (37.8 years for XL patients, 49.6 years for AR patients, Table 4), and even better figures are to be expected for the future, CGD remains a clinical challenge. Prophylactic treatment was not taken by a large percentage of the patients (Table 16). Adequate support, consisting of regular check-ups by a committed physician, prophylactic treatment and prompt intervention when infection is suspected remain the cornerstone of the treatment of CGD. The development of new therapeutical strategies, such as gene therapy, could also improve outcome [30], [37].

In conclusion, the many data collected from this cohort broadly confirm earlier assumptions on the clinical course and causative pathogens of CGD. Further investigations should be performed in a prospective manner, including data from more European countries.
